# Long‐term real‐world effectiveness of deucravacitinib in psoriasis: A 52‐week prospective study stratified by prior apremilast or biologic therapy

**DOI:** 10.1111/1346-8138.17665

**Published:** 2025-02-07

**Authors:** Teppei Hagino, Hidehisa Saeki, Eita Fujimoto, Naoko Kanda

**Affiliations:** ^1^ Department of Dermatology Nippon Medical School Chiba Hokusoh Hospital Inzai Japan; ^2^ Department of Dermatology Nippon Medical School Tokyo Japan; ^3^ Department of Dermatology Fujimoto Dermatology Clinic Funabashi Japan

**Keywords:** apremilast, biologic, deucravacitinib, long‐term, psoriasis, tyrosine kinase 2

## Abstract

Real‐world evidence on the long‐term effectiveness of deucravacitinib, a selective tyrosine kinase 2 inhibitor for psoriasis, remains limited, particularly in patients with different histories of systemic treatments. We evaluated the 52‐week effectiveness of deucravacitinib in patients with psoriasis, stratified by a history of apremilast or biologic usage. This prospective, single‐center study included 110 patients with moderate‐to‐severe psoriasis who received daily deucravacitinib (6 mg). Psoriasis Area and Severity Index (PASI) and Dermatology Life Quality Index (DLQI) scores during the treatment were analyzed in subgroups stratified by a history of apremilast or biologic usage. Deucravacitinib decreased PASI and DLQI scores for 52 weeks in psoriasis patients, both with and without prior apremilast or biologic usage. The percent reductions from baseline PASI or DLQI at week 52 were similar in apremilast‐experienced patients (92% or 77.9%) and apremilast‐naive patients (88.3% or 81.6%), respectively. The achievement rates of PASI 100 or absolute PASI ≤1 at week 52 in apremilast‐experienced patients (30.8% or 61.5%) were slightly higher than those in apremilast‐naive patients (20.5% or 46.2%). The percent reductions from baseline PASI or DLQI at week 52 in biologic‐naive patients (91.6 or 82.8%) were slightly higher than those in biologic‐experienced patients (57.6% or 63.6%), respectively. The achievement rates of PASI 75, 100 or absolute PASI ≤1 at week 52 in biologic‐naive patients (84.4%, 24.4%, or 53.3%) were slightly higher than those in biologic‐experienced patients (57.1%, 14.3%, or 28.6%), respectively. Deucravacitinib generated sustained 52‐week effectiveness in diverse patient subgroups, supporting its role as a universal treatment for psoriasis.

## 1 INTRODUCTION

Psoriasis is a chronic inflammatory skin disorder characterized by scaly, indurated erythema, and affects approximately 2%–4% of the population of Western countries or 0.3%–0.4% of the Japanese population.^1^, ^2^, ^3^, ^4^, ^5^ This disease is associated with substantially reduced quality of life (QoL), psychosocial distress, and stigma.^6^, ^7^, ^8^ Further, psoriasis patients are frequently associated with cardiovascular diseases, diabetes, or depression.^9^, ^10^

The main axis in the pathogenesis of psoriasis is the abnormal activation of interleukin (IL)‐23 and IL‐17 signaling pathways, and therapies targeting these cytokines have been developed and generated remarkable therapeutic effects.^11^, ^12^, ^13^, ^14^ However, novel therapies are still required that are safe, effective, and convenient for patients. Tyrosine kinase 2 (TYK2), a member of the Janus kinase (JAK) family, is regarded as a novel treatment target for psoriasis. This molecule transduces intracellular signals from IL‐12, IL‐23, and type I interferons (IFNs), involved in the pathogenesis of psoriasis.^15^ Deucravacitinib is an oral medicine that binds allosterically to the regulatory (pseudokinase) domain of TYK2, and highly specifically inhibits the activity of TYK2 with extremely less inhibition of JAK1, JAK2, and JAK3.^16^, ^17^, ^18^, ^19^ Owing to its inhibition of IL‐23‐ and type I IFN‐mediated signaling, deucravacitinib is considered to be a promising new treatment for psoriasis.^16^, ^17^

Previous phase III clinical trials have shown that deucravacitinib significantly improves skin rash and QoL in patients with psoriasis, with good tolerability.^20^, ^21^, ^22^, ^23^, ^24^, ^25^, ^26^ In addition, our 16‐week observational study found that deucravacitinib relieves ongoing symptoms, improves patient‐reported outcomes, and does not cause serious safety or tolerability problems.^27^ Psoriasis lesions in difficult‐to‐treat areas, such as the genital region, scalp, and nails, can seriously affect patients' daily life and social activities.^28^, ^29^, ^30^, ^31^ In our 24‐week follow‐up study focusing on these challenging sites, deucravacitinib improved symptoms of these sites and patients' QoL.^32^ These findings suggest that deucravacitinib is a promising oral treatment that can cure diverse symptoms and impaired QoL. However, long‐term (around 1‐year) real‐world data for deucravacitinib remain limited.^33^ In particular, little is known about its effectiveness for patients with prior usage of apremilast (an oral phosphodiesterase 4 inhibitor) or biologics. Since prior systemic therapies may influence the responsiveness to deucravacitinib treatment, long‐term real‐world data stratified by a history of systemic treatments are needed. Such information would be useful to identify the patients optimal for deucravacitinib treatment and may support personalized medicine for psoriasis patients. The aim of this study was to assess the 52‐week real‐world effectiveness of deucravacitinib (6 mg/day) in patients with psoriasis stratified by a history of apremilast or biologic usage. The study aimed to compare the long‐term therapeutic effects of deucravacitinib in patients with different treatment histories, and to enable more personalized medicine for psoriasis.

## 2 METHODS

### 2.1 Study design

This prospective, single‐center study was conducted from December 2022 to September 2024 in Japan. Patients aged ≥15 years with a clinical diagnosis of moderate‐to‐severe psoriasis were included. All the patients received oral deucravacitinib (6 mg once daily) for up to 52 weeks without concomitant topical agents. Prior to initiation of deucravacitinib treatment, some patients were treated with apremilast or biologics.

### 2.2 Data collection

Demographic and clinical characteristics were recorded at week 0, including age, sex, body mass index (BMI), disease duration, history of psoriatic arthritis, and involvement of scalp, nails, or the genital area. Comorbidities such as diabetes and cardiovascular diseases, smoking status, and previous systemic treatments (apremilast or biologics) were documented. Clinical indices assessed were the Psoriasis Area and Severity Index (PASI), and the Dermatology Life Quality Index (DLQI).

### 2.3 Inclusion criteria

This study included patients diagnosed with psoriasis vulgaris, psoriatic arthritis, or erythrodermic psoriasis. All participants received deucravacitinib (6 mg/day) for at least 4 weeks. Patients whose treatment was switched directly from apremilast or biologics to deucravacitinib experienced the switch without a washout period. Thus, for these patients, the PASI, and DLQI values at the time of switching (week 0) were defined as baseline values.

### 2.4 Exclusion criteria

Patients who received retreatment with deucravacitinib after discontinuation were excluded. Additionally, patients with the following characteristics were excluded: severe cardiovascular diseases (heart failure, myocardial infarction, or stroke), malignancies, active infections, known hypersensitivity to deucravacitinib or any of its components, and pregnancy or breastfeeding.

### 2.5 Assessments

Psoriasis Area and Severity Index and DLQI were assessed at weeks 0, 4, 16, 28, 40, and 52. The proportions of patients achieving PASI 75, 90, or 100 (≥75%, ≥90%, and 100% improvement from baseline PASI, respectively), as well as those achieving absolute PASI ≤1 or ≤2, were calculated at each time‐point. For patients with a baseline DLQI of ≥2, the proportions achieving DLQI 0/1 (score of 0 or 1 with a ≥2‐point reduction from baseline) were also determined simultaneously. All clinical outcomes were assessed in the total population and subgroups stratified by a history of apremilast or biologic usage.

### 2.6 Ethical considerations

This study was conducted in accordance with the Declaration of Helsinki and was approved by the institutional review board of the participating institution. Written informed consent was obtained from all the participants prior to enrollment.

### 2.7 Statistical analysis

Continuous variables with a normal distribution were presented as mean ± standard deviation (SD) while non‐normally distributed variables were expressed as median and interquartile range (IQR). The analyses were performed on an as‐observed basis. Because the baseline characteristics of each group differed (e.g., disease duration, age) and no placebo‐controlled design was implemented, conducting significance tests between apremilast‐experienced and biologic‐experienced groups could lead to invalid conclusions (such as biased *p*‐values). Therefore, we did not perform formal significance testing to avoid these statistical limitations. All statistical analyses were performed using Easy R (Jichi Medical University Saitama Medical Center).

## 3 RESULTS

### 3.1 Baseline characteristics

Among the total 110 patients, 71 were apremilast‐naïve and 39 were apremilast‐experienced, while 95 were biologic‐naive and 15 were biologic‐experienced (Table 1). Additionally, three patients had received both apremilast and biologic therapy prior to deucravacitinib treatment. Of the total patients, 53.6% had been treated with topical corticosteroids and/or vitamin D3 alone. The age and BMI of total patients were median (IQR) 63.0 (51.0–75.8) years and 23.6 (20.2–25.7) kg/m^2^, respectively. The disease duration was median (IQR) 7.0 (3.0–20.0) years. Plaque psoriasis was the most common type (84.5%), followed by psoriatic arthritis (10.9%), and erythrodermic psoriasis (4.5%); 41.8% of total patients were current smokers, and 4.5% had diabetes mellitus. The mean ± SD baseline PASI or DLQI scores in total patients was 14.5 ± 11.2 or 8.8 ± 5.4, respectively. All 39 apremilast‐experienced patients switched to deucravacitinib because of inadequate responses, whereas among the 15 biologic‐experienced patients, five switched because of inadequate responses, one owing to adverse events, and nine for economic reasons. Among these 15 biologic‐experienced patients, 13 had switched once, and two had switched twice before starting deucravacitinib.

**TABLE 1:** Baseline characteristics of patients stratified by a history of apremilast or biologic usage.

	Apremilast‐naïve (*n* = 71)	Apremilast‐experienced (*n* = 39)	Biologic‐naive (*n* = 95)	Biologic‐experienced (*n* = 15)	Total patients (*n* = 110)
Male sex	48 (67.6)	26 (66.7)	65 (68.4)	9 (60.0)	74 (67.3)
Age, median (IQR), years	63 (52–75.5)	66 (47.5–75.5)	61 (50.5–75.0)	73 (66.0–81.5)	63.0 (51.0–75.8)
BMI median (IQR), (kg m^−2^)	22.9 (20.1–24.2)	24.5 (22.1–27.6)	23.6 (20.3–25.4)	24.8 (19.9–27.1)	23.6 (20.2–25.7)
Disease duration median (IQR), years	6 (2–20)	8 (3–20)	6.0 (2.0–20.0)	13.5 (10.5–40.5)	7.0 (3.0–20.0)
Type of psoriasis					
Plaque psoriasis	57 (80.3)	36 (92.3)	82 (86.3)	11 (73.3)	93 (84.5)
Psoriatic arthritis	10 (14.1)	2 (5.1)	8 (8.4)	4 (26.7)	12 (10.9)
Erythrodermic psoriasis	4 (5.6)	1 (2.6)	5 (5.3)	0	5 (4.5)
Other characteristics					
Current smokers	32 (45.1)	14 (35.9)	40 (42.1)	6 (40.0)	46 (41.8)
Diabetes mellitus	5 (7.0)	2 (5.1)	5 (5.3)	0	5 (4.5)
Prior treatments					
Topical corticosteroid and/or vitamin D3 alone	59 (83.1)	0	59 (62.1)	0	59 (53.6)
Apremilast	0	39 (100)	36 (37.9)	3 (20.0)	39 (35.5)
Adalimumab	1 (1.4)	0	0	1 (6.7)	1 (0.9)
Bimekizumab	5 (7.0)	0	0	5 (33.3)	5 (4.5)
Ixekizumab	1 (1.4)	1 (2.6)	0	2 (13.3)	2 (1.8)
Guselkumab	4 (5.6)	0	0	4 (26.7)	4 (3.6)
Tildrakizumab	4 (5.6)	2 (5.1)	0	6 (40.0)	6 (5.5)
Baseline clinical indices					
DLQI, mean ± SD	9.4 ± 6.1	7.7 ± 3.4	9.2 ± 5.7	6.9 ± 2.8	8.8 ± 5.4
PASI, mean ± SD	15.0 ± 11.5	13.5 ± 10.8	15.5 ± 11.3	7.8 ± 8.0	14.5 ± 11.2

*Note*: Data are presented as *n* (%) unless otherwise indicated.

Abbreviations: BMI, body mass index; DLQI, Dermatology Life Quality Index; IQR, interquartile range; PASI, Psoriasis Area and Severity Index; SD, standard deviation.

### 3.2 Transition of PASI and DLQI in subgroups stratified by a history of apremilast usage during 52‐week treatment with deucravacitinib (Figure 1)

**FIGURE 1:**
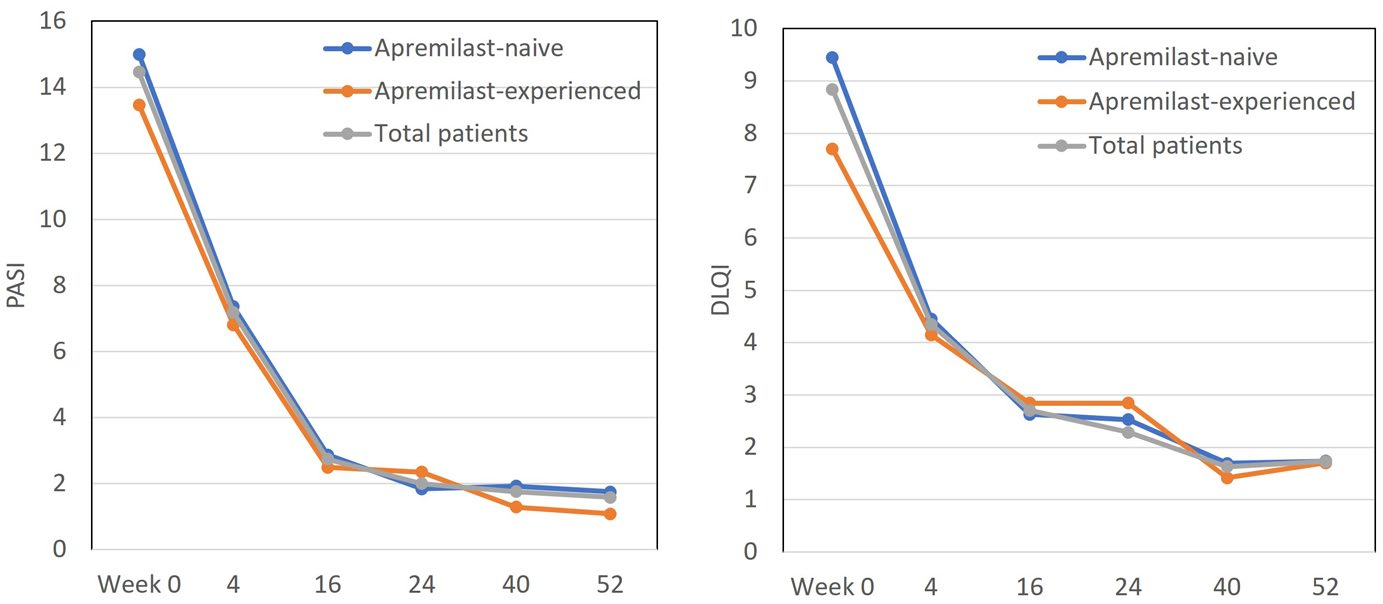
Transition of Psoriasis Area and Severity Index (PASI) and Dermatology Life Quality Index (DLQI) scores in psoriasis patients stratified by a history of apremilast usage during 52‐weeks’ treatment with deucravacitinib. Data are mean scores for apremilast‐naive patients (blue), apremilast‐experienced patients (orange), and total patients (gray).

The mean PASI score in apremilast‐naive and ‐experienced patients continued to decrease in similar magnitudes from week 0 to week 52; from 14.99 to 1.75 (88.3% reduction) in apremilast‐naive patients, and from 13.47 to 1.08 (92% reduction) in apremilast‐experienced patients, respectively, with a corresponding decrease from 14.46 to 1.58 (89.1% reduction) in total patients. The mean DLQI score continued to decrease in the two subgroups in similar magnitudes from week 0 to week 52; from 9.45 to 1.74 (81.6% reduction) in apremilast‐naive patients, and from 7.70 to 1.70 (77.9% reduction) in apremilast‐experienced patients, with a corresponding decrease from 8.84 to 1.73 (80.4% reduction) in total patients. These findings indicate that deucravacitinib improved skin rash and QoL in similar magnitudes in apremilast‐naive and ‐experienced patients throughout the treatment period.

### 3.3 Transition of rates achieving PASI 75, 90, 100, absolute PASI ≤2, PASI ≤1, and DLQI 0/1 in subgroups stratified by a history of apremilast usage, during 52‐weeks’ treatment with deucravacitinib (Figure 2)

**FIGURE 2:**
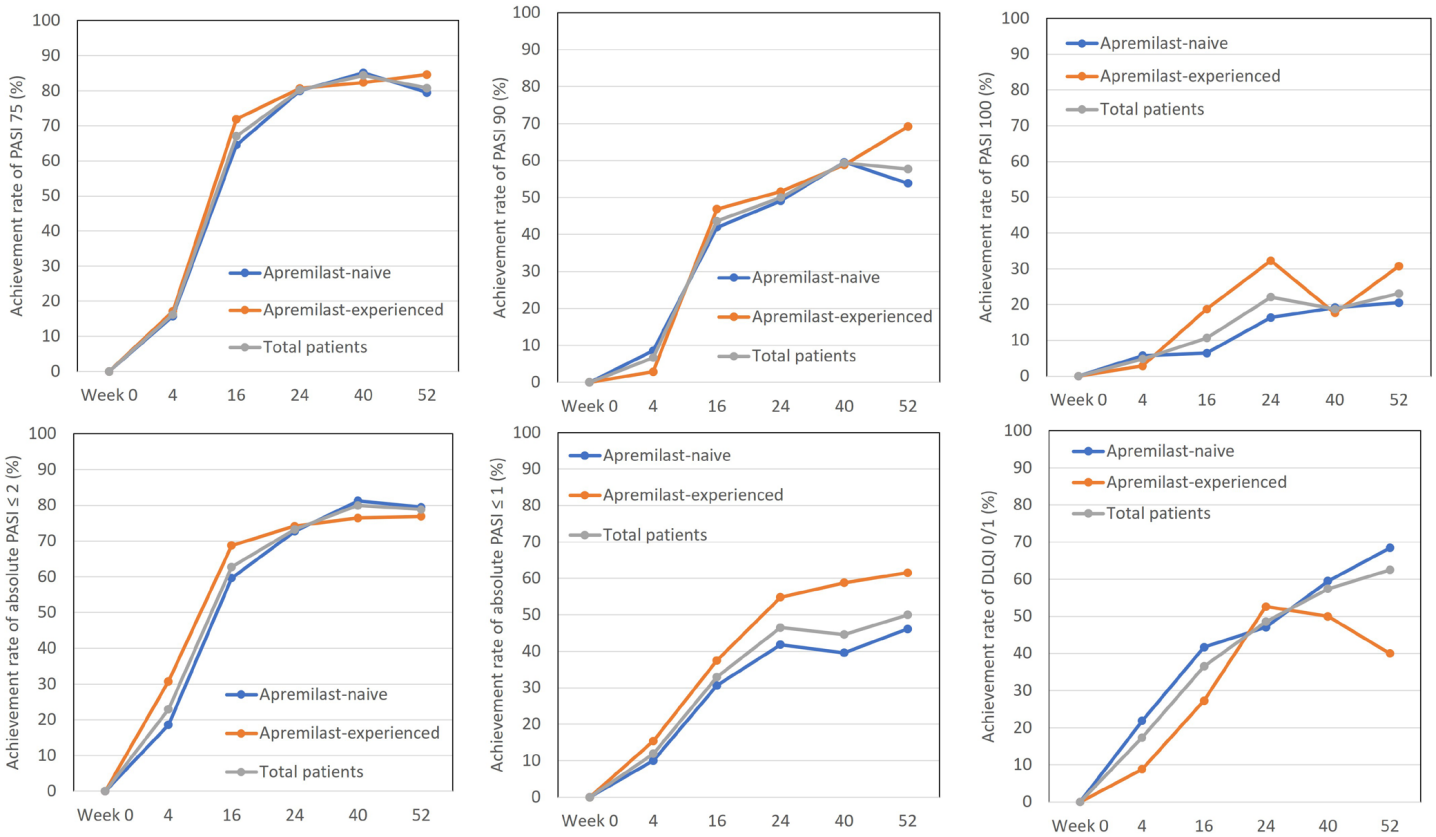
Transition of rates achieving Psoriasis Area and Severity Index (PASI) 75, PASI 90, PASI 100, absolute PASI ≤2, absolute PASI ≤1, or Dermatology Life Quality Index (DLQI) 0/1 in psoriasis patients stratified by a history of apremilast usage, during 52‐weeks’ treatment with deucravacitinib. Data shown are the achievement rates for apremilast‐naive patients (blue), apremilast‐experienced patients (orange), and total patients (gray).

In apremilast‐naive and ‐experienced patients, achievement rates of PASI 75, absolute PASI ≤2, and DLQI 0/1 similarly continued to increase until week 52; achievement rates of PASI 75 and absolute PASI ≤2 at week 52 were 79.5% and 79.5% in apremilast‐naive patients versus 84.6% and 76.9% in apremilast‐experienced patients. On the other hand, achievement rates of PASI 90, PASI 100, and absolute PASI ≤1, stricter end‐points, continued to increase until week 52 in both groups, although the magnitude of increase in the apremilast‐experienced group appeared slightly higher; at week 52, achievement rates of PASI 90, PASI 100, and absolute PASI ≤1 were 53.9%, 20.5%, and 46.2% respectively in apremilast‐naive patients, and 69.2%, 30.8%, and 61.5% respectively in apremilast‐experienced patients. In apremilast‐naive patients, the achievement rate of DLQI 0/1 continuously increased from 21.88% to 68.42% (from week 4 to 52), and similarly from 21.9% to 62.5% in total patients. On the other hand, in apremilast‐experienced patients, the achievement rate of DLQI 0/1 increased from 8.82% to 52.63% (from week 4 to 24), followed by a slight decrease to 40% at week 52.

### 3.4 Transition of PASI and DLQI in subgroups stratified by a history of biologic usage, during 52‐weeks’ treatment with deucravacitinib (Figure 3)

**FIGURE 3:**
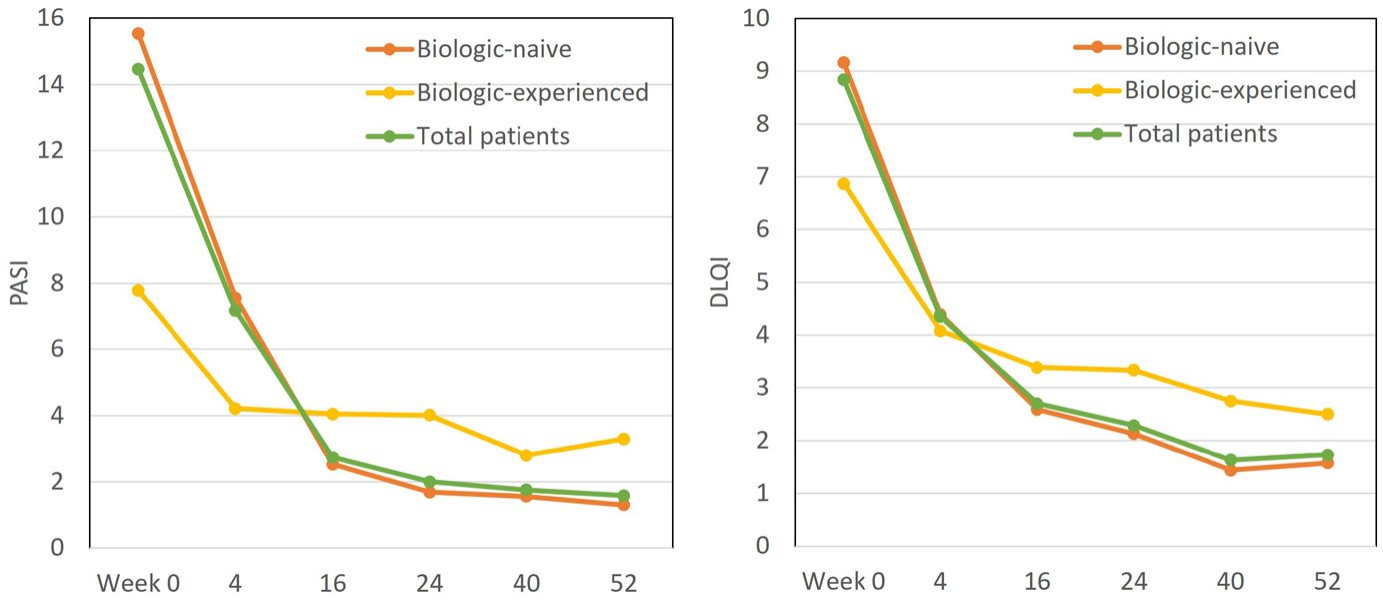
Transition of Psoriasis Area and Severity Index (PASI) and Dermatology Life Quality Index (DLQI) scores in psoriasis patients stratified by a history of biologic usage, during 52‐weeks’ treatment with deucravacitinib. Data are mean scores for biologic‐naive patients (orange), biologic‐experienced patients (yellow), and total patients (green).

Both in biologic naive and experienced patients, mean PASI scores continued to decrease from week 0 to week 52; however, the magnitude of decrease appeared higher in biologic‐naive patients: from 15.53 to 1.30 in biologic‐naive patients (91.6% reduction) versus from 7.78 to 3.30 (57.6% reduction) in biologic‐experienced patients. Similarly, both in biologic naive and experienced patients, mean DLQI scores steadily decreased from week 0 to week 52; however, the magnitude of decrease appeared higher in biologic‐naive patients: from 9.16 to 1.58 (82.8% reduction) in biologic‐naive patients versus from 6.87 to 2.50 (63.6% reduction) in biologic‐experienced patients.

### 3.5 Transition of rates achieving PASI 75, 90, 100, absolute PASI ≤2, absolute PASI ≤1, and DLQI 0/1 in subgroups stratified by a history of biologic usage, during the 52‐week treatment period with deucravacitinib (Figure 4)

**FIGURE 4:**
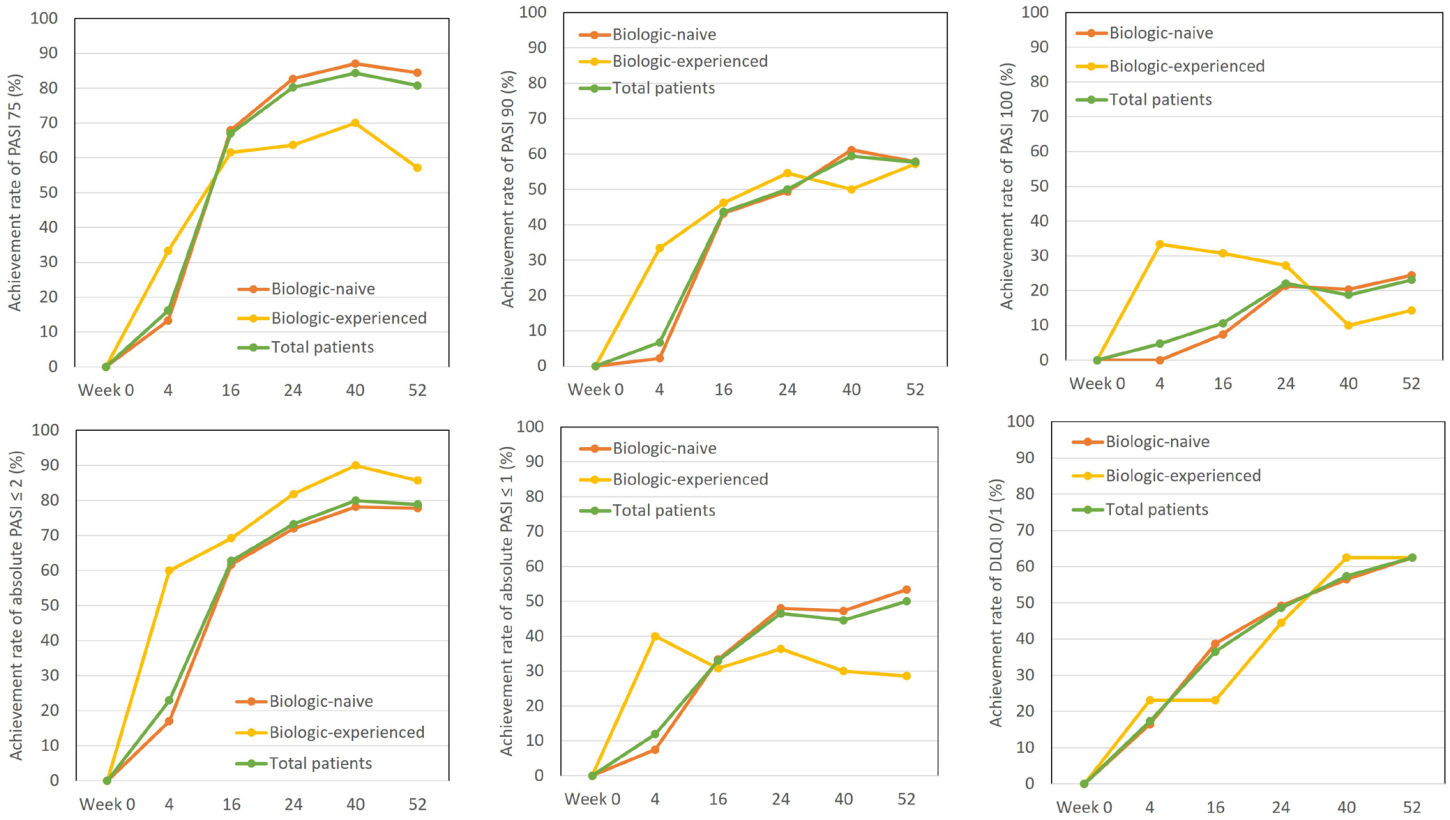
Transition of rates of achieving Psoriasis Area and Severity Index (PASI) 75, PASI 90, PASI 100, absolute PASI ≤2, absolute PASI ≤1, or Dermatology Life Quality Index (DLQI) 0/1 in psoriasis patients stratified by a history of biologic usage, during 52‐weeks’ treatment with deucravacitinib. Data shown are the achievement rates for biologic‐naive patients (orange), biologic‐experienced patients (yellow), and total patients (green).

Both in biologic‐naive and ‐experienced patients, achievement rates of PASI 75, PASI 90, absolute PASI ≤2, and DLQI 0/1 mostly continued to increase until week 52. At week 52, the achievement rates of PASI 75, PASI 90, and absolute PASI ≤2 were 84.4%, 77.8%, and 77.8% respectively in biologic‐naive patients, and 57.1%, 57.4%, and 85.7% respectively in biologic‐experienced patients. The biologic‐naive patients showed a higher achievement rate of PASI 75 at later treatment phases (weeks 24 to 52) compared to biologic‐experienced patients, whereas biologic‐experienced patients showed higher achievement rates of absolute PASI ≤2 throughout the 52‐week period compared to biologic‐naive patients. On the other hand, achievement rates of PASI 100 and absolute PASI ≤1, stricter endpoints, continued to increase until week 52 in biologic‐naive patients while these peaked at week 4 and plateaued or declined thereafter in biologic‐experienced patients. The achievement rates of PASI 100 and absolute PASI ≤1 at week 52 in biologic‐naive patients (24.4% and 53.3% respectively) appeared higher than those in biologic‐experienced patients (14.3% and 28.6% respectively). In both biologic‐naive and experienced patients, the achievement rate of DLQI 0/1 similarly continued to increase from week 4 to week 52; from 16.5% to 62.5% in biologic‐naive patients versus from 23.1% to 62.5% in biologic‐experienced patients.

## 4 DISCUSSION

In our study, apremilast‐experienced patients did not show significant differences in the reduction of PASI and DLQI scores compared with apremilast‐naive patients (Figure 1). Previous phase III clinical trials have also reported that deucravacitinib can be effective even in patients who have had an inadequate response to apremilast.^20^ Our present results also indicate that the prior apremilast usage may not attenuate the clinical outcomes of deucravacitinib treatment.

Furthermore, apremilast‐experienced patients showed slightly higher achievement rates of PASI 100 or absolute PASI ≤1, rather strict endpoints, at treatment phases later than week 24 compared with apremilast‐naive patients (Figure 2). These findings indicate that prior treatment with apremilast may cooperatively act to maximize the effects of deucravacitinib on skin clearance. On the other hand, the rate achieving DLQI 0/1 declined at treatment phases later than week 40 in apremilast‐experienced patients (Figure 2), though the mean DLQI score was similarly reduced in apremilast‐naive and ‐experienced patients (Figure 1). The reason for the late phase decline should be further clarified by precisely analyzing the transition of scores of individual DLQI items.

Though mean PASI and DLQI scores continued to decrease from week 0 to week 52 in both biologic‐naive and ‐experienced patients, these scores at week 52 were higher and their percent reduction was lower in biologic‐experienced patients than in biologic‐naive patients (Figure 3), indicating a lower responsiveness to deucravacitinib treatment in biologic‐experienced patients. The rates achieving PASI ≤2 at all treatment phases were higher, and rates achieving PASI 75, 90, 100, or absolute PASI ≤1 at treatment phases earlier than week 16 appeared higher in biologic‐experienced patients than in biologic‐naive patients (Figure 4), possibly because baseline PASI scores were lower in the former group. However, at later phases, the achievement rates of PASI 75, 90, 100, or absolute PASI ≤1 in biologic‐naive patients reached or surpassed those in biologic‐experienced patients (Figure 4), possibly reflecting the limited responsiveness to deucravacitinib in the latter. Our findings indicate that biologic‐naive patients may generate higher responsiveness to deucravacitinib treatment than biologic‐experienced patients, indicating that the former is a more suitable population for deucravacitinib treatment. Similarly, a previous study reported that biologic‐naive patients could achieve a higher response rate to deucravacitinib, which supports our observation.^24^

In line with this finding, a network meta‐analysis of systemic therapies for psoriasis reported that deucravacitinib showed lower short‐, mid‐, and long‐term PASI 75 achievement rates compared to IL‐23 and IL‐17 inhibitors (e.g., risankizumab, ixekizumab).^34^ In addition, the biologic‐experienced patients in our study had a longer disease duration (median [IQR] 60 [2.0–20.0] vs 13.5 [10.5–40.5] years), suggesting a more refractory status. A previous study indicated that patients with shorter disease duration (<2 years) achieved higher clearance rates with IL‐23p19 antibodies (e.g., risankizumab, guselkumab) than those with longer disease durations,^35^, ^36^ which may partially explain the difference in responsiveness. However, it remains unclear whether a longer disease duration also reduces the responsiveness to TYK2 inhibitors. Although TYK2 inhibitors and IL‐23p19 antibodies have different mechanisms of action, both ultimately target the IL‐23 pathway.

These results may be possible because prior biologic treatment might modulate the patients' immune system and affect the steric structure of TYK2 or its affinity for deucravacitinib. Since this was not a clinical trial, patients previously treated with biologics were directly switched to deucravacitinib without clear washout periods. Further studies should set a clear washout period for biologic‐experienced patients to accurately evaluate clinical outcomes. Alternatively, five out of 15 biologic‐experienced patients in this study experienced treatment failures to biologics, and this population might potentially have poorer responsiveness to treatments for psoriasis generally. One recent study specifically investigated 14 patients with at least two biologic therapy failures and reported that 64% achieved partial or complete responses to deucravacitinib, with an average response time of 2.6 months. Although that study involved a small sample size and short follow‐up period, the authors concluded that deucravacitinib may be a feasible treatment option even for patients with multiple systemic treatment failures.^37^ Further studies using a larger cohort should evaluate the responsiveness to deucravacitinib in patients with biologic therapy failures. In addition, biologic‐experienced patients in this study accounted for 17.2% of total patients, a rather limited population, and were of older age with higher rate of arthritis compared to biologic‐naive patients (median [IQR] 73 [66.0–81.5] vs 61 (50.5–75.0) years; 26.7% vs 8.4%), which may influence the treatment responsiveness. Further studies should be conducted using a cohort with a higher proportion of biologic‐experienced patients, and the results should be adjusted for confounding factors such as age or presence of arthritis.

There are several limitations in this study. First, the study was conducted at a single institution. Second, only Japanese patients were included. Third, the number of biologic‐experienced patients was small. Fourth, we did not stratify patients by specific comorbidities, the types of biologics previously used, or the number of biologic switches. Last, because we did not perform formal statistical testing due to baseline differences and lack of a control group, our comparisons between subgroups (e.g., apremilast‐experienced vs apremilast‐naive, biologic‐experienced vs biologic‐naive) are descriptive only, and differences should be interpreted with caution. This was an observational study without a control group, which limits the ability to draw universal conclusions from the findings.

## 5 CONCLUSION

In summary, deucravacitinib reduced PASI and DLQI scores throughout the 52‐week treatment period for psoriasis patients, both in apremilast‐naive and ‐experienced subgroups, and in biologic‐naive and ‐experienced subgroups, indicating the improvement of skin rash and QoL irrespective of prior usage of these treatments. The magnitude of reduced PASI and DLQI was not significantly different between apremilast‐naive and apremilast‐experienced patients while it appeared slightly lower in biologic‐experienced patients than in biologic‐naive patients.

## CONFLICT OF INTEREST STATEMENT

Teppei Hagino, Hidehisa Saeki, and Naoko Kanda received lecture fees from Bristol Myers Squibb. The other authors have no conflicts of interest to declare.

Hidehisa Saeki is an Editorial Board member of *The Journal of Dermatology* and a co‐author of this article. To minimize bias, he was excluded from all editorial decision‐making related to the acceptance of this article for publication.

## ETHICS STATEMENT

The study was conducted in accordance with the Declaration of Helsinki and approved by the Ethics Committee of Nippon Medical School Chiba Hokusoh Hospital (protocol code and approval: H‐2023‐095, December 7, 2023).

## Data Availability

The datasets used and/or analyzed during the current study are available from the corresponding author upon reasonable request.
